# Supplementary material for: Live imaging of alveologenesis in precision-cut lung slices reveals dynamic epithelial cell behaviour
Source: Nat Commun. 2019 Mar 12;10:1178. doi: 10.1038/s41467-019-09067-3 (PMC6414680; doi:10.1038/s41467-019-09067-3)
Supplement: Supplementary file 1 — Supplementary Information [file 41467_2019_9067_MOESM1_ESM.pdf]

Supplementary Information

Live imaging of alveologenesis in precision-cut lung slices reveals dynamic epithelial cell behaviour.

Akram et al.

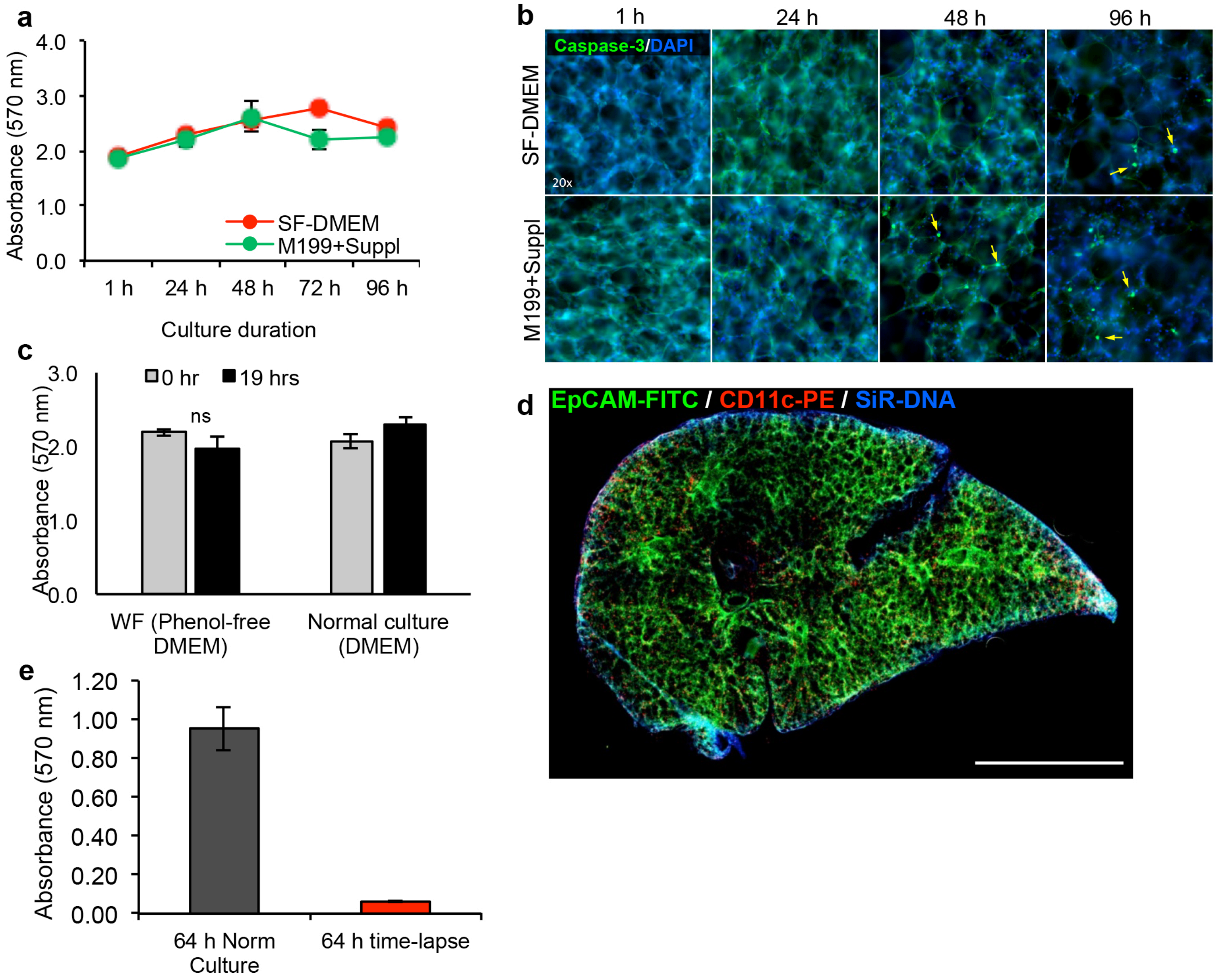

Supplementary figure 1: Optimising PCLS culture conditions.
MTT cell viability assay on adult lung PCLS cultured in serum free (SF) DMEM or supplemented M199 media for up to 96 h **(a)**, n = 3 PCLS per condition, per timepoint. Widefield images of Nucview 488 Caspase-3 (green) and DAPI, nuclear (blue) staining of live adult lung PCLS cultured in SF-DMEM, top panel or supplemented M199 media, bottom panel, for up to 96 h **(b)**, n = 3 PCLS per condition, per timepoint; yellow arrows indicate cleaved caspase-3 +ve cells. MTT cell viability assay on adult lung PCLS at t=0 and after time-lapse imaging for 19 h in phenol-free DMEM or normal culture in phenol red-containing DMEM **(c)**, n=3 PCLS per condition, per timepoint. ns = not significant, one-way ANOVA with Tukey's post hoc test. Deconvolved widefield single plane z-stack image of an entire P3 PCLS labelled with EpCAM-FITC (green), CD11c-PE (red) and SiR-DNA (blue) **(d)**. MTT cell viability assay on P3 PCLS following 64 hours normal or time-lapse culture **(e)**, n=3 PCLS per group. Scale bar = 1000 μm. Error bars are defined as s.e.m.

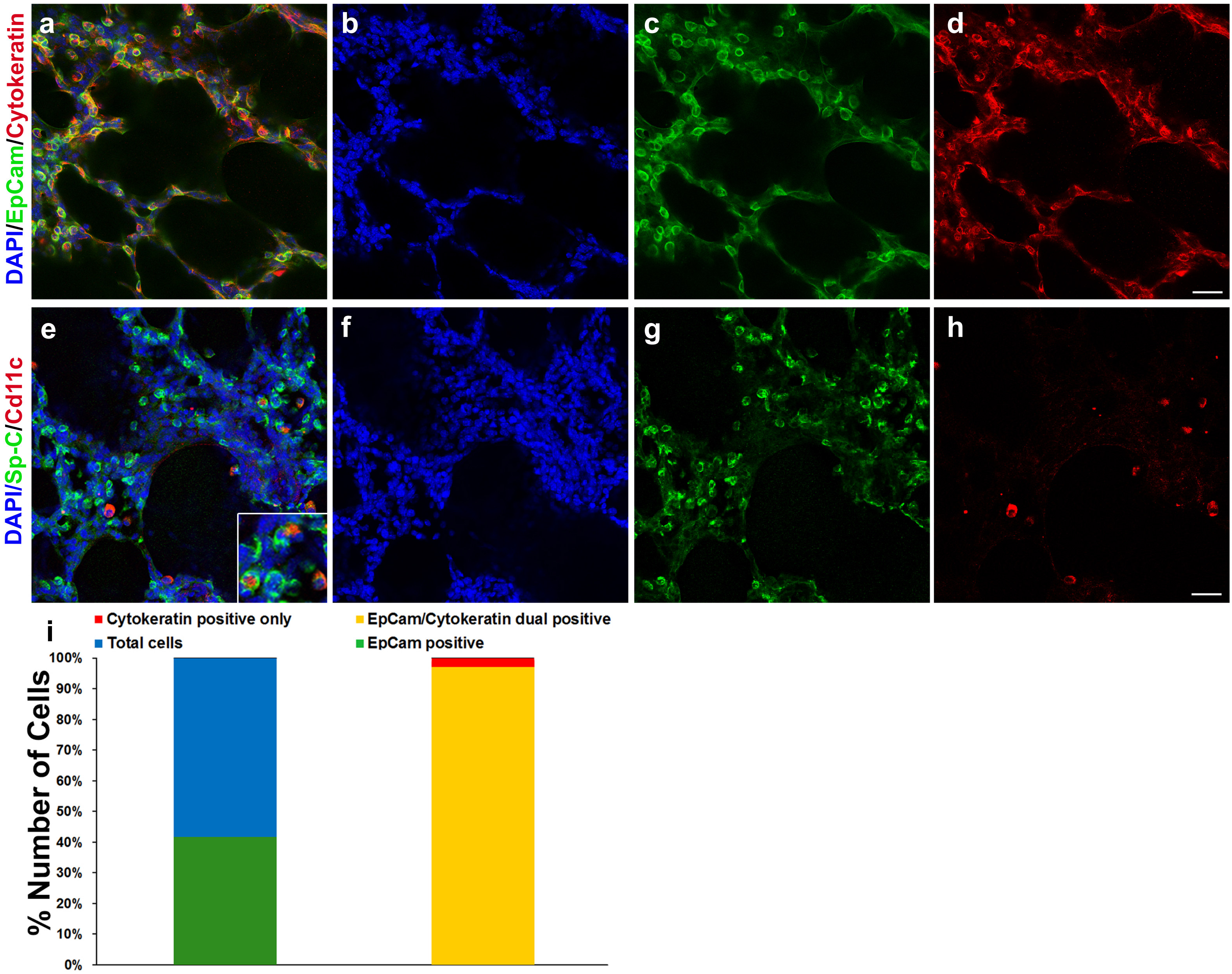

Supplementary figure 2: Dual EpCAM-FITC/Cytokeratin and SP-C/CD11c immunostaining on P3 PCLS.

Confocal images of P3 PCLS labelled with DAPI (blue) **(a,b)**, EpCAM-FITC (green) **(a,c)** and Cytokeratin (red) **(a,d)**. Dual EpCAM and Cytokeratin positive cells can be observed in **(a)**. Confocal images of P3 PCLS labelled with DAPI (blue) **(e,f)**, Sp-C (green) **(e,g)** and CD11c-PE (red) **(e,h)**. Dual Sp-C and CD11c staining can be observed in a small minority of cells (**insert in e**). Percentage of EpCAM positive (green), Cytokeratin positive (red) and dual EpCAM/Cytokeratin positive cells (yellow), in P3 PCLS **(i),** n=2 independent experiments with 3 PCLS for each set of dual antibody staining per experiment. Scale bar = 50 µm.

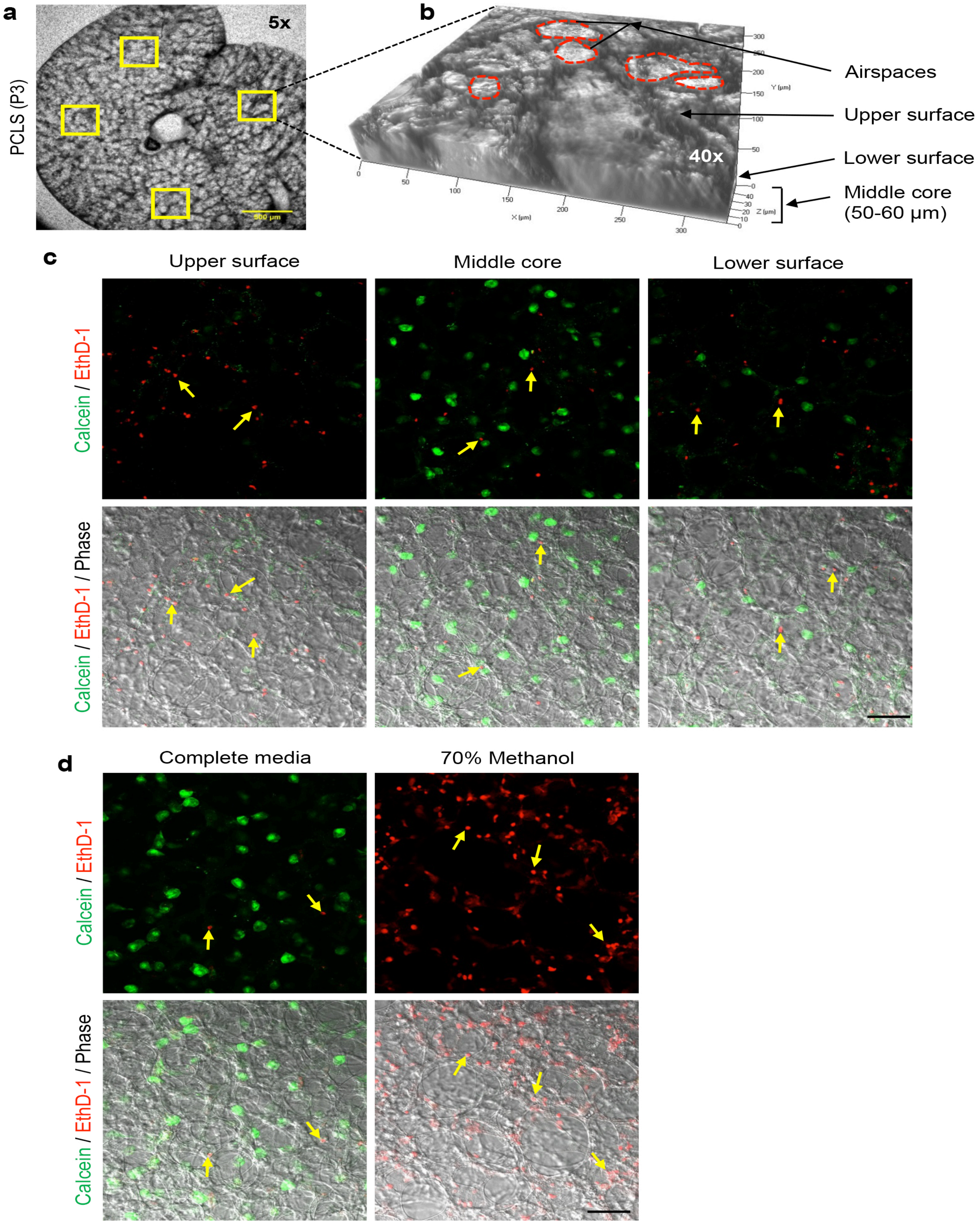

Supplementary figure 3: Details of widefield PCLSi.

Widefield time-lapse videos were obtained from four fields (yellow squares) of peripheral lung in each PCLS using a 40x objective **(a)**, Scale bar = 500 µm. 3-D image of a 300 µm PCLS; z-stacks were obtained from 4 separate fields within a 50-60 µm central core of each PCLS at regular intervals throughout each time-lapse experiment **(b)**, red lines indicate examples of alveolar airspaces. Confocal images of adult PCLS labelled with calcein (live cells, green) and ethidium (dead cells, red) dyes from upper surface, middle core and lower surface of PCLS **(c)**. Confocal images of adult PCLS labelled with calcein and ethidium cultured in normal culture medium (live cell control), or 70% methanol (dead cell control) **(d)**. Yellow arrows indicate dead cells. Scale bar = 50 µm (c, d).

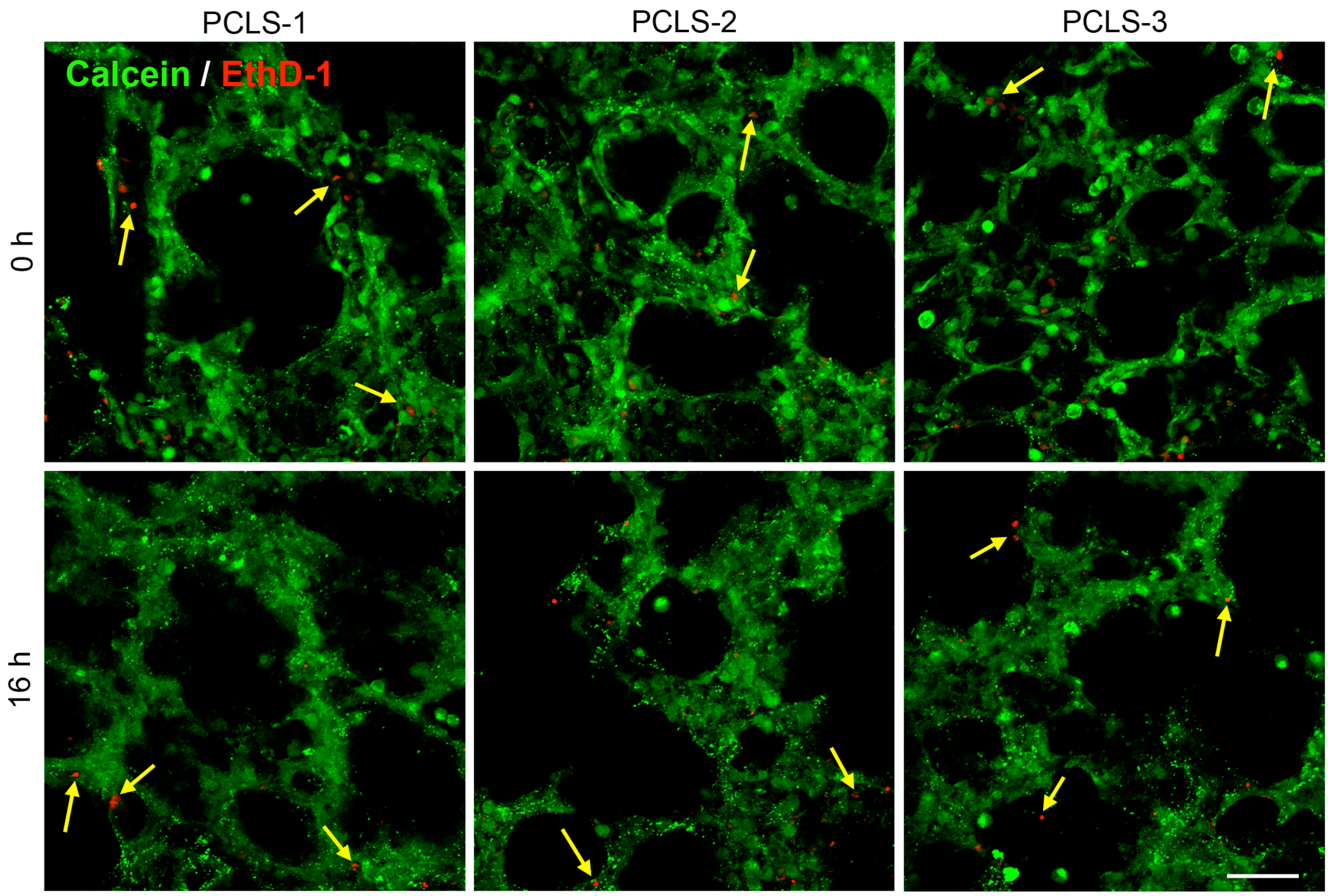

Supplementary figure 4: Live/Dead assay on P3 PCLS after 16 hours of live imaging.

Confocal images of P3 PCLS labelled with calcein (green, live cells) and ethidium (red, dead cells) dyes at 0 hour and after 16 hours of live imaging, n=3 PCLS per timepoint. Yellow arrows indicate ethidium positive dead cells. Scale bar =50 µm.

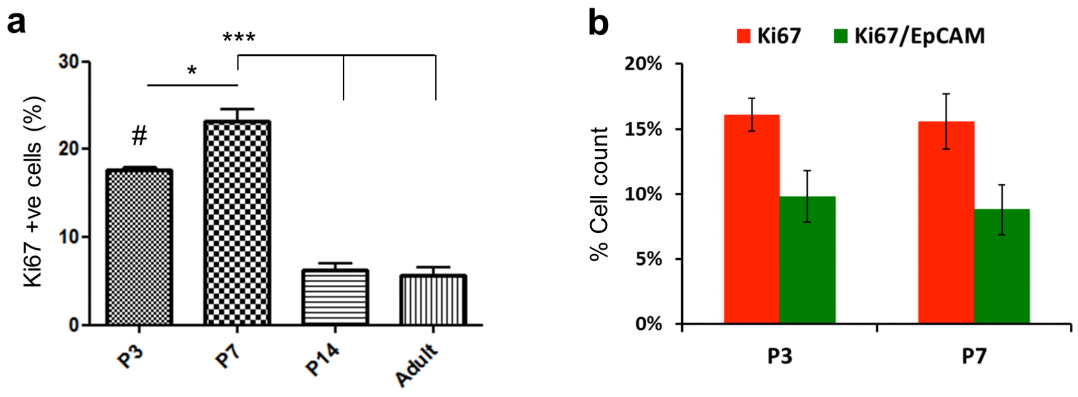

Supplementary figure 5: Total cell proliferation in vivo and total and epithelial cell specific proliferation in ex vivo PCLS.

Graph showing the percentage of Ki67 positive cells in P3, P7, P14 and adult lung sections by immunohistochemistry **(a)**, n=3 individual mice per age group. 3 H&E sections were quantified per mouse, per age group. *p<0.05, **p<0.001, #p<0.05 vs. P7, one-way ANOVA with Tukey's post hoc test. Graph showing the percentage of Ki67 positive proliferating cells at P3 and P7 (red) and the percentage of EpCAM and Ki67 dual positive cells (green) at P3 and P7 in PCLS by immunofluorescence **(b)**, n=2 independent experiments on 2 separate mice with quantification obtained from 3 PCLS per experiment. Error bars are defined as s.e.m.

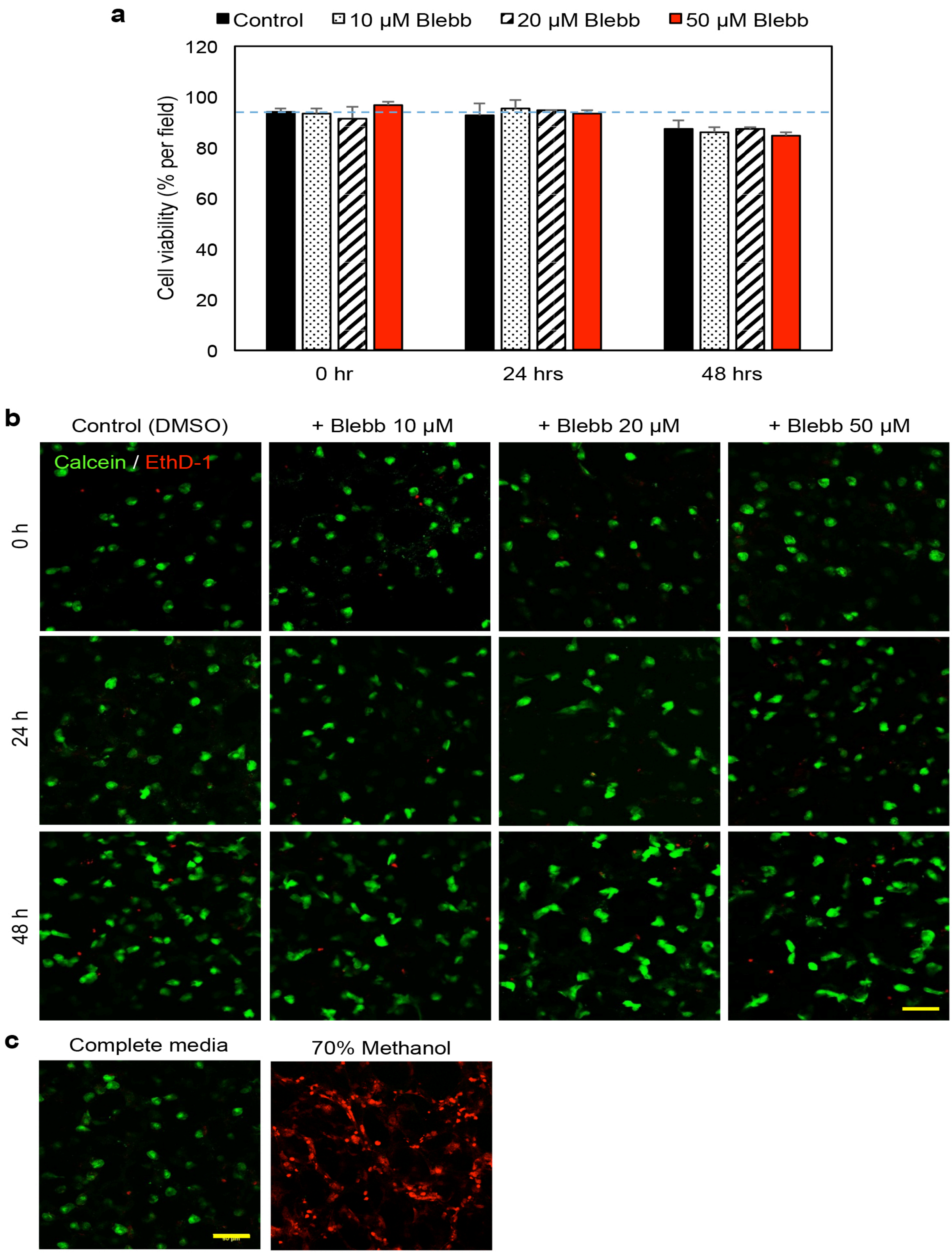

Supplementary figure 6: Dose response of blebbistatin in adult PCLS.
Percentage of live cells in adult PCLS treated with DMSO control media or with 10, 20 and 50 µM blebbistatin following 0, 24 and 48 hours of culture **(a)**, viability was determined by labelling PCLS with calcein and ethidium. Confocal images of adult PCLS treated with DMSO control media or with blebbistatin (10, 20, 50 µM) following 0, 24 and 48 hours of culture and labelled with calcein (live cells, green) and ethidium (dead cells, red) dyes **(b)**. PCLS treated with normal culture medium (live cell control), or 70% methanol (dead cell control), bottom panels **(c)**. n=2 independent experiments using 2 separate mice, with duplicate slices per group per experiment **(a, b, c)**. Scale bars = 50 µm. Error bars are defined as s.e.m.

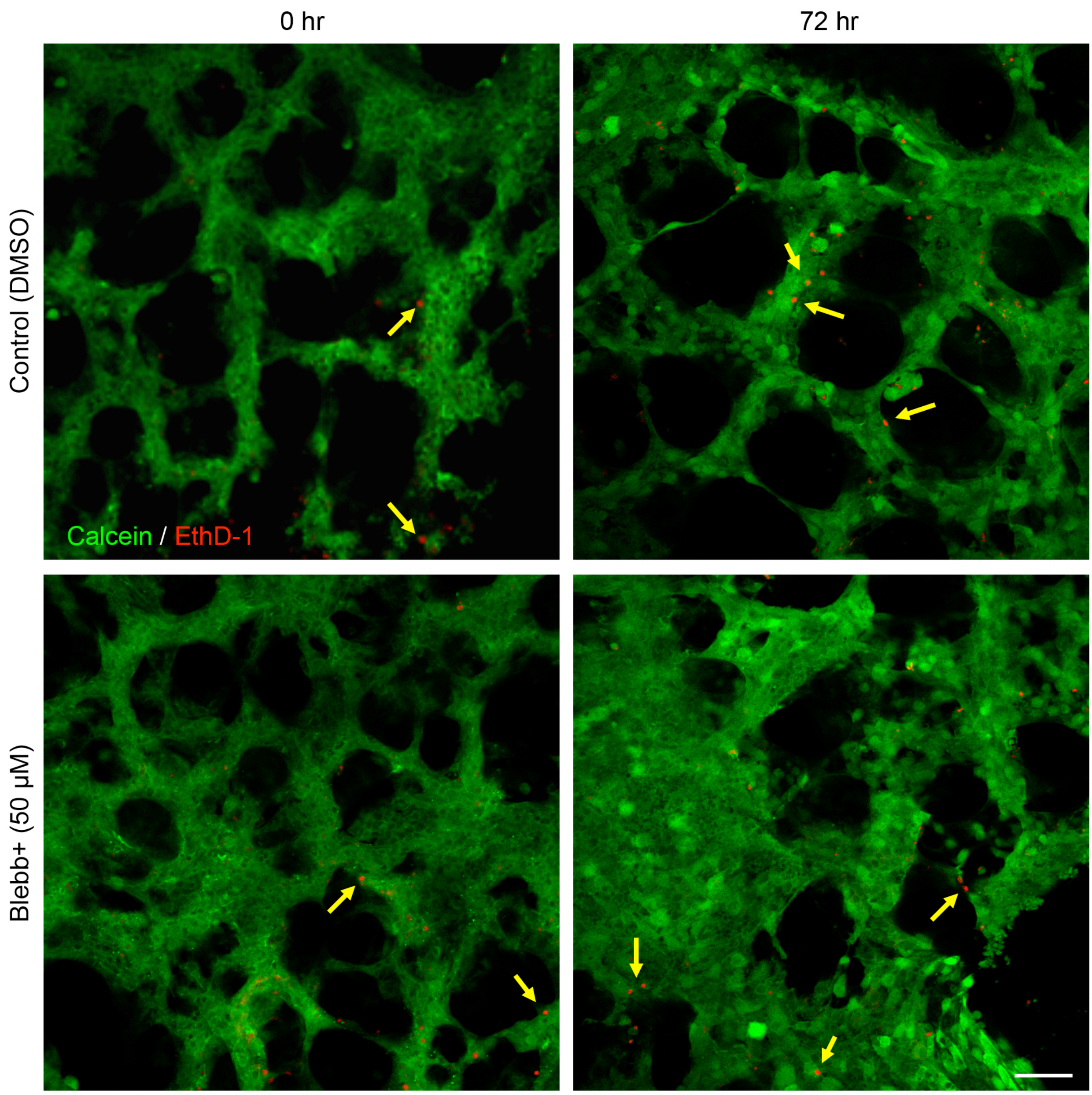

Supplementary figure 7: Live/Dead assay on P3 PCLS cultured for 72 hours in presence or absence of blebbistatin.

Confocal images of P3 PCLS labelled with calcein (green, live cells) and ethidium (red, dead cells) dyes treated with DMSO control or 50µM blebbistatin following 0 or 72 hours of culture. Yellow arrows indicate dead cells. n=3 independent experiments using 3 separate mice, with duplicate slices per condition per experiment. Scale bar = 50 µm.

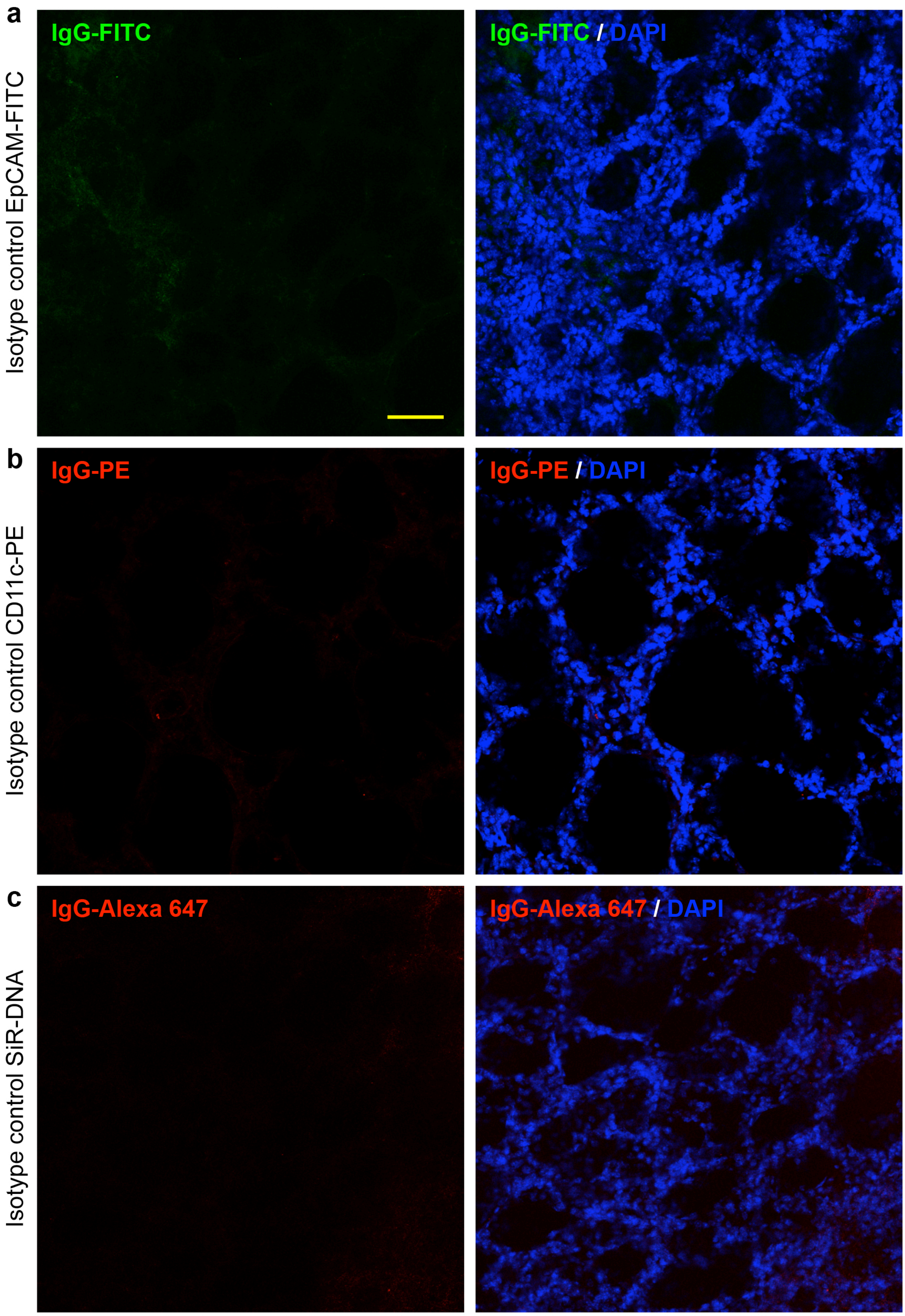

Supplementary figure 8: Isotype control immunofluorescent staining on P3 PCLS

Confocal images of P3 PCLS labelled with IgG-FITC (green) and DAPI (blue) **(a)**, IgG-PE (red) and DAPI (blue) **(b)**, IgG-Alexa 647 (red) and DAPI (blue) **(c)**. Scale bar = 50 μm.
