# Supplementary material for: Live imaging of alveologenesis in precision-cut lung slices reveals dynamic epithelial cell behaviour
Source: Nat Commun. 2019 Mar 12;10:1178. doi: 10.1038/s41467-019-09067-3 (PMC6414680; doi:10.1038/s41467-019-09067-3)
Supplement: Supplementary file 3 — Description of Additional Supplementary Files [file 41467_2019_9067_MOESM3_ESM.docx]

**Description of Supplementary Files**

**File Name:** **Supplementary Movie 1**

**Description:** Long-term PCLSi Brightfield (A) and EpCAM-FITC (B) video of P3 PCLS imaged for 64 hours at 60-minute intervals. a = airspaces.

**File Name:** **Supplementary Movie 2**

**Description:** PCLSi pre- and post deconvolution Raw (A) and deconvolved (B) videos of P3 PCLS labelled with EpCAM-FITC (green) and SiR-DNA (magenta), imaged for 16 hours at 15 minute intervals. a= airspaces.

**File Name:** **Supplementary Movie 3**

**Description:** Epithelial cell movement in P3 and P7 PCLS EpCAM-FITC labelled PCLS from P3 (A) and P7 (B) mice imaged for 8 hours at 15 minute intervals. a= airspaces.

**File Name:** **Supplementary Movie 4**

**Description:** Epithelial cell movement in P14 and adult PCLS EpCAM-FITC labelled PCLS from P14 (A) and adult (B) mice imaged for 8 hours at 15 minute intervals. a= airspaces.

**File Name:** **Supplementary Movie 5**

**Description:** Septation, cell clustering and epithelial cell migration of epithelial cells to existing airspaces in P3 PCLS (A)Epithelial cell dynamics in P3 PCLS. EpCAM-FITC (green) and SiR-DNA (magenta) labelled P3 PCLS imaged for 12 hours 45 minutes at 15 minute intervals. Red arrows indicate migrating septa, as one existing airway subdivides into two (a1-a2 and a3-a4). Blue circles indicate areas where cell clustering can be seen. Migrating epithelial cells intercalate with existing alveolar wall epithelial cells (yellow arrows) around two airspaces, a5 and a6. (B) Zoomed in version of video 5A, septation 1. EpCAM-FITC (green) and SiRDNA (magenta) labelled P3 PCLS imaged for 12 hours 45 minutes at 15 minute intervals. Zoomed in version of video 5A, focusing on a migrating septum subdividing airspaces a1 and a2. (Ci) Zoomed in version of video 5A, septation 2. EpCAM-FITC (green) and SiRDNA (magenta) labelled P3 PCLS imaged for 12 hours 45 minutes at 15 minute intervals. Zoomed in version of video 5A, focusing on a migrating septum subdividing airspaces a3 and a4. (D) Zoomed in version of video 5A, cell clustering. EpCAM-FITC (green) and SiR-DNA (magenta) labelled P3 PCLS imaged for 12 hours 45 minutes at 15 minute intervals. Zoomed in version of blue-circled areas in video 5A where cell clustering can be observed. (E) Zoomed in version of video 5A, cell migration. EpCAM-FITC (green) and SiR-DNA (magenta) labelled P3 PCLS imaged for 12 hours 45 minutes at 15 minute intervals. Zoomed in 3D render of video 5A showing migrating epithelial cells as they intercalate with existing epithelial cells lining 2 airspaces a5 and a6.

**File Name:** **Supplementary Movie 6**

**Description:** Epithelial cells integrate into an existing airspace EpCAM-FITC (red) and SiR-DNA (cyan) labelled P3 PCLS imaged for 14 hours 15 minutes at 15 minute intervals. Epithelial cells 1,2 and 3 (green arrows) migrate towards an existing alveolar airspace (white circle). a= airspaces.

**File Name:** **Supplementary Movie 7**

**Description:** Hollowing in P3 PCLS Brightfield video of P4 PCLS imaged for 18 hours at 15 minute intervals. Red arrowheads indicate an area of lung parenchyma in which an opening or hollow appears; yellow dotted lines outline adjacent airspaces. a= airspaces.

**File Name: Supplementary Movie 8**

**Description:** Raw video of hollowing in EpCAM labelled PCLS Raw video showing EpCAM-FITC (red) and SiR-DNA (cyan) labelled P3 PCLS imaged for 14 hours 45 minutes at 15 minute intervals. White circles outline two areas (A and B) of lung parenchyma where holes (hollows) open up. a= airspaces.

**File Name: Supplementary Movie 9**

**Description:** Deconvolved video of hollowing in EpCAM labelled PCLS. Deconvolved version of video 8 showing EpCAM-FITC (red) and SiR-DNA (cyan) labelled P3 PCLS imaged for 14 hours 45 minutes at 15 minute intervals. White circles outline two areas of lung parenchyma (A and B) where holes (hollows) open up. a= airspaces.

**File Name: Supplementary Movie 10**

**Description:** Cell extension around an existing airway wall Brightfield video of a P3 PCLS imaged for 16 hours at 10 minute intervals. Yellow dotted line marks the outer perimeter of the airway at t=0. Yellow arrow= starting point of the cell, red arrow = end point of the cell, white dashed arrow indicates the direction of cell extension. a= airspaces.

**File Name: Supplementary Movie 11**

**Description:** Cell extension in EpCAM labelled PCLS Zoomed in greyscale version of video 5A showing the reverse view of video 5Ci in which cell extension of an EpCAM positive epithelial cell can be seen around an alveolar wall, bottom left. P3 PCLS imaged for 12 hours 45 minutes at 15 minute intervals. White arrow indicates the region where the extending cell can be seen.

**File Name: Supplementary Movie 12**

**Description:** Visualisation of the epithelium and capillary network in P3 PCLS. EpCAM-FITC (green) and PECAM-Alexa 647 (red) labelled P3 PCLS imaged for 12 hours 30 minutes at 15 minute intervals. Both EpCAM positive epithelial and PECAM positive endothelial cells can be seen in an extending septum during septation.

**File Name: Supplementary Movie 13**

**Description:** Zoomed in version of video 12. EpCAM-FITC (green) and PECAM-Alexa 647 (red) labelled P3 PCLS imaged for 12 hours 30 minutes at 15 minute intervals. Zoomed in version of video 12 where both epithelial and endothelial cells can be observed in an extending septum.

**File Name: Supplementary Movie 14**

**Description:** Control and blebbistatin treated P3 PCLS EpCAM-FITC (green) and SiR-DNA (magenta) labelled P3 PCLS treated with DMSO control media (A), imaged for 14 hours at 15 min intervals or 50µM blebbistatin containing media (B), imaged for 14 hours 15 minute intervals. a= airspaces.

**File Name: Supplementary Movie 15**

**Description:** Control and Cytochalasin D treated P3 PCLS EpCAM-FITC (green) and SiR-DNA (magenta) labelled P3 PCLS treated with control (A) or 100 ng/ml cyto-D containing (B) media, imaged for 14 hours at 15 minute intervals. a= airspaces.
