# Supplementary material for: Live imaging of alveologenesis in precision-cut lung slices reveals dynamic epithelial cell behaviour
Source: Nat Commun. 2019 Mar 12;10:1178. doi: 10.1038/s41467-019-09067-3 (PMC6414680; doi:10.1038/s41467-019-09067-3)
Supplement: Supplementary file 23 — Reporting Summary [file 41467_2019_9067_MOESM23_ESM.pdf]

nature research

Corresponding author(s): Charlotte Dean

Last updated by author(s): Jan 22, 2019

Reporting Summary

Nature Research wishes to improve the reproducibility of the work that we publish. This form provides structure for consistency and transparency in reporting. For further information on Nature Research policies, see Authors & Referees and the Editorial Policy Checklist.

Statistics

For all statistical analyses, confirm that the following items are present in the figure legend, table legend, main text, or Methods section.

n/a	Confirmed	
☐	☒	The exact sample size (n) for each experimental group/condition, given as a discrete number and unit of measurement
☐	☒	A statement on whether measurements were taken from distinct samples or whether the same sample was measured repeatedly
☐	☒	The statistical test(s) used AND whether they are one- or two-sided<br>*Only common tests should be described solely by name; describe more complex techniques in the Methods section.*
☐	☐	A description of all covariates tested
☐	☒	A description of any assumptions or corrections, such as tests of normality and adjustment for multiple comparisons
☐	☒	A full description of the statistical parameters including central tendency (e.g. means) or other basic estimates (e.g. regression coefficient) AND variation (e.g. standard deviation) or associated estimates of uncertainty (e.g. confidence intervals)
☐	☒	For null hypothesis testing, the test statistic (e.g. F, t, r) with confidence intervals, effect sizes, degrees of freedom and P value noted<br>*Give P values as exact values whenever suitable.*
☒	☐	For Bayesian analysis, information on the choice of priors and Markov chain Monte Carlo settings
☒	☐	For hierarchical and complex designs, identification of the appropriate level for tests and full reporting of outcomes
☒	☐	Estimates of effect sizes (e.g. Cohen's d, Pearson's r), indicating how they were calculated

Our web collection on statistics for biologists contains articles on many of the points above.

Software and code

Policy information about availability of computer code

Data collection	Zen acquisition software (Zeiss)
Data analysis	GraphPad Prism version 5, Excel (Office 2011), Icy open source software version 1.9.8.0, Huygens Esential version 17.10, Fiji ImageJ 2.0, NIS-Elements software (version 4.50, Nikon Instruments)

For manuscripts utilizing custom algorithms or software that are central to the research but not yet described in published literature, software must be made available to editors/reviewers. We strongly encourage code deposition in a community repository (e.g. GitHub). See the Nature Research guidelines for submitting code & software for further information.

Data

Policy information about availability of data

All manuscripts must include a data availability statement. This statement should provide the following information, where applicable:

- Accession codes, unique identifiers, or web links for publicly available datasets
- A list of figures that have associated raw data
- A description of any restrictions on data availability

The data that support the findings of this study are available either in the manuscript or supplementary files. Any additional data from this study are available from the corresponding author upon reasonable request.

Field-specific reporting

Please select the one below that is the best fit for your research. If you are not sure, read the appropriate sections before making your selection.

☒ Life sciences ☐ Behavioural & social sciences ☐ Ecological, evolutionary & environmental sciences

For a reference copy of the document with all sections, see nature.com/documents/nr-reporting-summary-flat.pdf

Life sciences study design

All studies must disclose on these points even when the disclosure is negative.

Sample size	sample sizes were chosen based on our knowledge from previous studies i.e. the sample sizes used were sufficient to enable statistically meaningful data to be obtained. Details of sample sizes for each experiment are provided either in the methods section or are shown as individual data points in graphs.
Data exclusions	N/A
Replication	the number of replicates performed for each experiment (n number) are included in the legend for each figure.
Randomization	No randomization was used.
Blinding	Blinding was not used in this study as it was not relevant in most of the experiments. Except for acquisition of mean linear intercept and airspace counts in Figs 2,7 and 8 where the investigator was blind to the groupings during data acquisition.

Reporting for specific materials, systems and methods

We require information from authors about some types of materials, experimental systems and methods used in many studies. Here, indicate whether each material, system or method listed is relevant to your study. If you are not sure if a list item applies to your research, read the appropriate section before selecting a response.

Materials & experimental systems

n/a	Involved in the study	
☐	☒	Antibodies
☒	☐	Eukaryotic cell lines
☒	☐	Palaeontology
☐	☒	Animals and other organisms
☒	☐	Human research participants
☒	☐	Clinical data

Methods

n/a	Involved in the study	
☒	☐	ChIP-seq
☒	☐	Flow cytometry
☒	☐	MRI-based neuroimaging

Antibodies

Antibodies used	All antibodies used in this study are available commercially. Details of the supplier name, catalogue number and clone name of antibodies are provided in the methods section of the manuscript.
Validation	Details of validation for each antibody are provided on the manufacturers websites. Additional validation of antibodies in this study are shown in Supplemental figure 8.

Animals and other organisms

Policy information about studies involving animals; ARRIVE guidelines recommended for reporting animal research

Laboratory animals	C57/BL6 female time mated mice were used for adult timepoints and male and female pups from these mice were used for all other timepoints (P3, P7, P14).
Wild animals	N/A
Field-collected samples	N/A
Ethics oversight	All animal maintenance and procedures were carried out according to the requirements of the animal scientific procedures act 1986. Ethical approval for animal work in this study was obtained from the South Kensington and St Mary's AWERB committee.

Note that full information on the approval of the study protocol must also be provided in the manuscript.
